# Phase I study of intratumoral injection of talimogene laherparepvec for the treatment of advanced pancreatic cancer

**DOI:** 10.1093/oncolo/oyae200

**Published:** 2024-12-02

**Authors:** Karie Runcie, Yadriel Bracero, Avishai Samouha, Gulam Manji, Helen E Remotti, Tamas A Gonda, Yvonne Saenger

**Affiliations:** Department of Hematology, Oncology New York Presbyterian Hospital, New York, NY 10032, United States; Department of Oncology Albert Einstein College of Medicine, Bronx, NY 10461, United States; Department of Oncology Albert Einstein College of Medicine, Bronx, NY 10461, United States; Department of Hematology, Oncology New York Presbyterian Hospital, New York, NY 10032, United States; Department of Anatomic Pathology, New York Presbyterian Hospital, New York, NY 10032, United States; NYU Grossman School of Medicine, New York, NY 10016, United States; Department of Oncology Albert Einstein College of Medicine, Bronx, NY 10461, United States

**Keywords:** pancreatic cancer, immunotherapy, talimogene laherparepvec, T-VEC, endoscopic treatment

## Abstract

**Background:**

Pancreatic ductal adenocarcinoma (PDAC) presents a redoubtable challenge due to late-stage diagnosis and limited treatment options, necessitating innovative therapeutic strategies.

**Methods:**

Here, we report our results investigating the safety and efficacy of talimogene laherparepvec (T-VEC), an FDA-approved oncolytic herpes simplex virus type 1, in patients with advanced PDAC. Nine patients with treatment-refractory advanced PDAC received escalating doses of T-VEC via endoscopic injection.

**Results:**

While no objective responses were observed, stable disease was achieved in 44% of patients, with a median overall survival of 7.8 months, including one patient who survived 28 months. Adverse events were manageable, with the maximum tolerated dose determined to be 10^8^ PFU/mL.

**Conclusion:**

Our findings underscore the feasibility of intratumoral T-VEC administration in advanced PDAC. Further investigation, particularly in combination with immunotherapies administered systemically is warranted to more optimally assess T-VEC in this challenging malignancy.

ClinicalTrials.gov Identifier: NCT03086642.

## Teaser

Results of a study investigating the safety and efficacy of talimogene laherparepvec in patients with advanced pancreatic ductal adenocarcinoma are reported.

**Lessons Learned**

- Oncolytic viruses can be safely administered endoscopically to patients with pancreatic cancer.
- Repeat endoscopic interventions confer an elevated risk of infection and should be considered in future trials.
- Although limited by a small sample size, 44% of patients achieving stable disease in a highly aggressive malignancy is promising and warrants further investigation.
- Observed local control of injected lesions and a high stable disease rate suggest that benefit may be achievable with combination systemic therapies.

## Discussion

Pancreatic ductal adenocarcinoma (PDAC) has one of the lowest 5-year survival rates highlighting the need for novel therapeutic approaches. This study evaluated talimogene laherparepvec (T-VEC), the only FDA-approved oncolytic virus for melanoma, as a treatment for patients with advanced PDAC. T-VEC was administered endoscopically via intratumoral injections to patients who had progressed on at least one systemic therapy. Eligible patients received up to 4 doses of T-VEC. The primary objective of the study was to determine the maximum tolerated dose (MTD) for T-VEC in patients with advanced pancreatic cancer.

**Figure 1.**
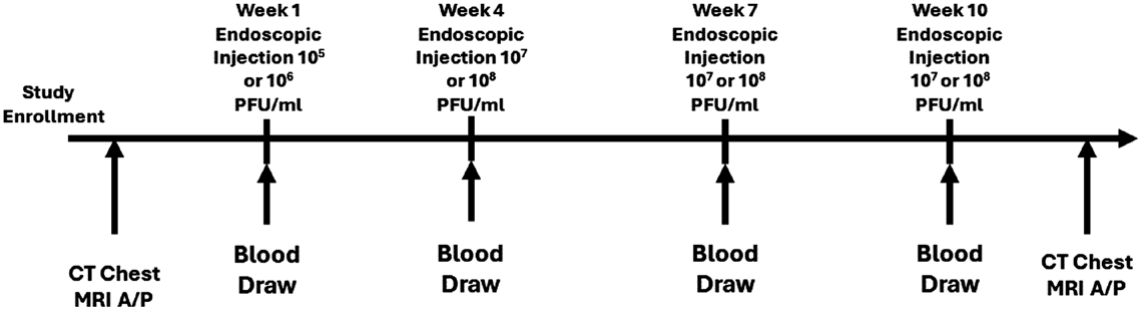
Study enrollment.

Nine patients enrolled in this open-label phase I dose escalation study from November 2017 to July 2021 (Figure 1). The MTD was determined to be the 10^8^ PFU/mL dose level consistent with prior studies in melanoma. Five patients exhibited stable disease. The overall survival rate was 67% at 6 months and 15% at 12 months. Importantly, no serious toxicities were directly linked to T-VEC, although 2 out of 9 patients experienced intra-abdominal infections requiring hospitalization. These infections were attributed to the injection procedure rather than the virus itself and were managed with intravenous antibiotics. This rate of complications aligns with previous reports of similar issues arising from endoscopic ultrasound (EUS)–administered viral therapies.

These findings are consistent with prior trials of oncolytic viral therapy in PDAC, which have typically resulted in the stabilization of locally injected lesions. Despite the induction of T-cell infiltration by oncolytic viruses, listeria, and radiation, a correlation between this infiltration and systemic response has yet to be established. While T-cell infiltration is probably necessary, it alone does not appear to be sufficient for achieving therapeutic benefit. The observed local control of injected lesions and high rates of stable disease with oncolytic therapy suggest potential benefits from combining these therapies with other systemic treatments.

One notable case from the study involved a patient who survived for more than 24 months. RNA analysis of this patient’s blood peripheral mononuclear cells (PBMCs) indicated upregulation of genes associated with autoimmune disease and cytokine signaling. This finding is in line with previous research suggesting that favorable immune changes in peripheral blood may correlate with better outcomes in patients with PDAC undergoing immunotherapy.

In conclusion, this study demonstrates that intratumoral injection of T-VEC in advanced patients with PDAC is generally safe and may contribute to stabilization of disease. These results highlight the potential for combining local oncolytic viral therapies with other systemic treatments to enhance therapeutic efficacy. Given the urgent need for effective PDAC treatments, further trials investigating such combination regimens are warranted.

## Trial Information

**Table:** 

Disease	Pancreatic cancer
Stage of disease/treatment	4
Prior therapy	At least one line of prior therapy
Type of study	Phase I, dose escalation (study design shown in Figure 2; dose escalation shown in Table 1)
Primary endpoint	Maximum tolerated dose
Secondary endpoints	Objective response rate and overall survival

## Drug Information

**Table:** 

Generic/working name	Talimogene laherparepvec
Company name	Amgen Inc
Drug type	Oncolytic virus
Dose	4.0 mL of 10^6^ PFU/mL to 4.0 mL of 10^8^ PFU/mL
Unit	PFU/mL
Route	Endoscopic injection
Schedule of administration	Four 21-day cycles

## Patient Characteristics

**Table:** 

Number of patients	
Male	6
Female	3
Stage	4
Age, median (range)	71 years
Ethnicity	
Hispanic	1 (11%)
Non-Hispanic	7 (78%)
Unknown	1 (11%)
Race	
White	6 (67%)
Black	1 (11%)
Asian	1 (11%)
Other	1 (11%)
Number of prior systemic therapies	
1	3 (22%)
2	5 (55%)
3	2 (22%)
Number of prior systemic therapies, median (range)	2 (2)
Performance status: ECOG	
0	5
1	4
2	0
3	0
4	0
Cancer types or histologic subtypes	Pancreatic adenocarcinoma, 9

## Primary Assessment Method

**Table:** 

Number of patients screened	10
Number of patients enrolled	9
Number of patients evaluable for toxicity	9
Number of patients evaluated for efficacy	9
Evaluation method	RECIST 1.1
Response assessment	
SD	4 (44%)
PD	5 (56%)
(Median) Duration assessment, PS	7.8 months
Duration of treatment	1.85 (0.7-2.3) months

## Outcome notes

### Safety and tolerability

**Figure 2.**
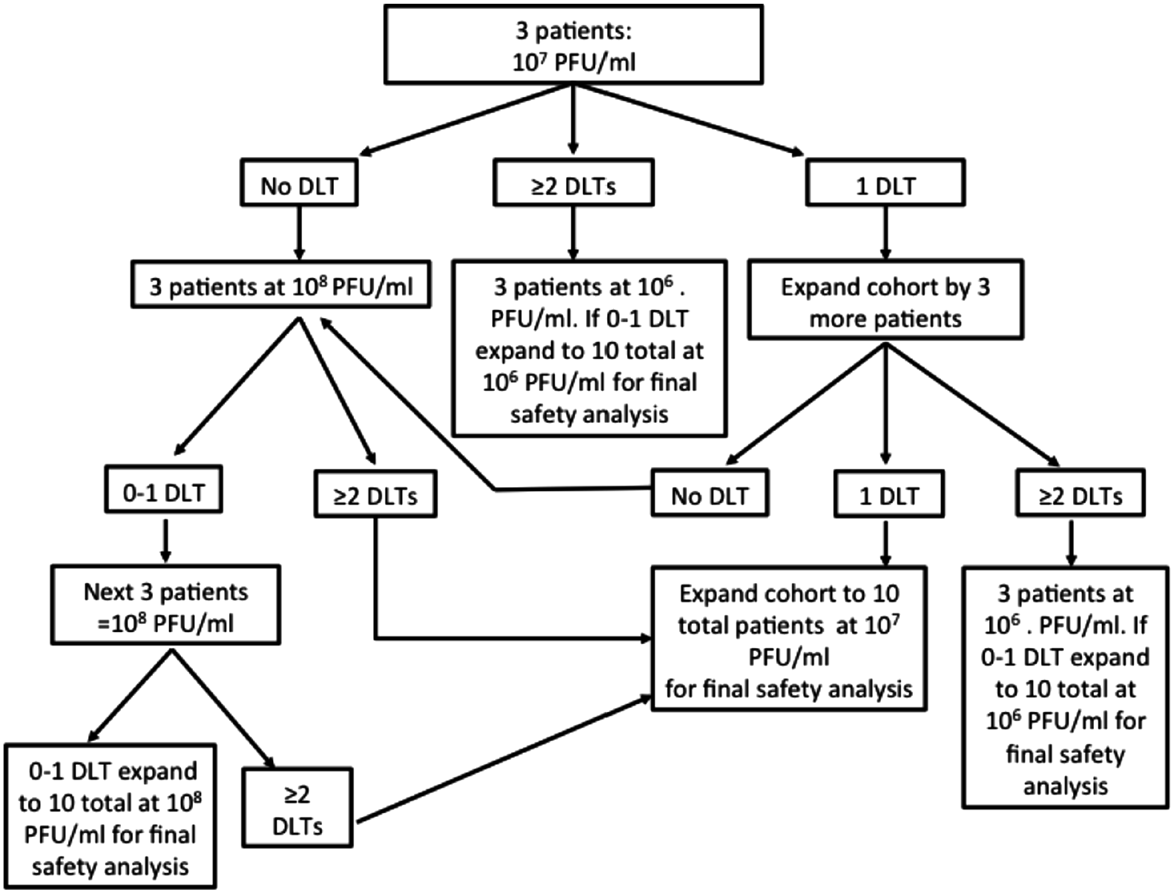
3 + 3 study design with cohort expansion to define the maximum tolerated dose (MTD). All patients received a week 1 test dose of 10^6^ PFU/followed by treatment doses at weeks 4, 7, and 10. 1 DLT was recorded in the first 3 patients, 0 DLTs in the second 3 patients, and 1 DLT in the last 3 patients enrolled.

Six patients received the 10^7^ PFU/mL dose level and 3 patients received the 10^8^ PFU/mL dose level due to dose escalation after 1 dose limiting toxicity was observed in 6 treated patients at the 10^7^ PFU dose (Table 1 and Figure 2). Eight of the 9 patients experienced treatment-related adverse events with fevers, chills, and intra-abdominal infection being the most common causes of adverse events (Table 2). Two patients had a grade 3 or 4 treatment-related adverse events due to intra-abdominal infection. Six of the 9 patients received all 4 doses of T-VEC; however, one patient only received 2 doses due to hospitalization for perisplenic abscess versus necrotic tumor and 2 patients received 3 doses, one due to hospitalization for pulmonary embolism and the other due to hospitalization for cholecystitis. Fatigue, dizziness, diarrhea, nausea, abdominal pain, and transaminitis were less common and occurred in 2 patients. The MTD was determined to be the 10^8^ PFU/mL dose level, equivalent to melanoma.^1^

**Table 1.** Dose escalation.

Dose level	Number of injections	Dose	Number of patients	Total treatments
1	4	4.0 mL of 10^7^ PFU/mL^*^	6	18
1	4	4.0 mL of 10^8^ PFU/mL	3	9

^*^For each dose, the first dose was 2 logs lower than the treatment dose, followed by 3 doses at the treatment dose to account for patients not previously exposed to HSV.

**Table 2.** Treatment-related adverse events and dose limiting toxicities.

	*n* = 9		
	Any grade, *n* (%)	Grade 1 or 2, *n* (%)	Grade 3 or 4, *n* (%)
Chills	5 (55.5)	5 (55.5)	0
Thromboembolic event	3 (33.3)	2 (22.2)	1 (11.1)
Fever	3 (33.3)	3 (33.3)	0
Nausea	2 (22.2)	1 (11.1)	0
Dizziness	2 (22.2)	2 (22.2)	0
Abdominal pain	2 (22.2)	2 (22.2)	0
Fatigue	2 (22.2)	2 (22.2)	0
Palpitations	1 (11.1)	1 (11.1)	0
Intra-abdominal infection	6 (66.6)	4 (44.4)	2 (22.2)^*^
Diarrhea	2 (22.2)	1 (11.1)	0
Alanine aminotransferase increased	2 (22.2)	1 (11.1)	0
Alkaline phosphatase increased	1 (11.1)	1 (11.1)	0
Aspartate aminotransferase increased	1 (11.1)	1 (11.1)	0
Edema limbs	1 (11.1)	1 (11.1)	0

^*^Dose limiting toxicity.

### Efficacy

**Figure 3.**
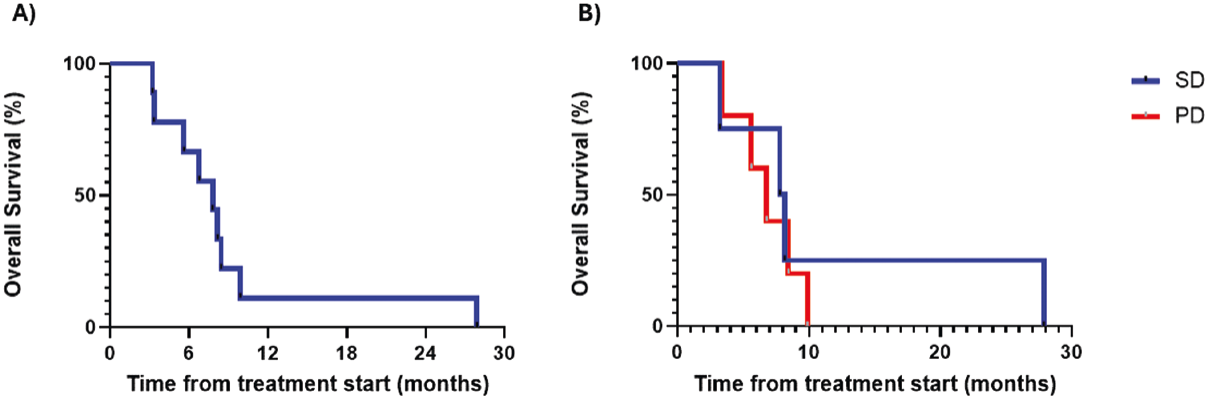
Kaplan-Meier overall survival curve for patients with pancreatic carcinoma post T-vec treatment. (A) Overall survival (in months) after treatment began. (B) Progressive free survival (in months) in stable patients versus disease-progressed patients.

The overall rate of stable disease at 3 months by RECIST v 1.1 was 44% in this population with very advanced disease with 4 patients having stable disease and 5 patients’ progressive disease including 2 patients who did not receive all 4 doses of oncolytic viral therapy. No objective response was noted. Median overall survival was 14.6 months in the 4 patients who had stable disease as compared with 6.8 months in patients who had disease progression. The median overall survival was 67% at 6 months and 15% at 12 months (Figure 3). One patient who achieved stable disease survived 28 months.

### Peripheral blood analysis

NanoString Analysis was performed on PBMCs obtained from all patients at study visits. Results for the patient who lived much longer than the others was compared and pathway analysis was performed (Table 3). Most differentially expressed pathways included T- and B-cell signaling and the most differentially expressed genes included Th1 genes TBX1, Thy1.1, CD8, and CCL5 (Tables 3 and 4).

**Table 3.** Differentially expressed pathways in long-term survivor.

	Count	Percent	*P*-value
Hematopoietic cell lineage	9	19.1	1.00E−08
Inflammatory bowel disease	7	14.9	3.80E−07
B-cell receptor signaling pathway	7	14.9	1.80E−06
Th17 cell differentiation	6	12.8	1.10E−04
Primary immunodeficiency	4	8.5	6.40E−04
Cell adhesion molecules	6	12.8	6.70E−04
Th1 and Th2 cell differentiation	5	10.6	7.40E−04
NF-kappa B signaling pathway	5	10.6	1.20E−03
Cytokine-cytokine receptor interaction	7	14.9	2.00E−03
Rheumatoid arthritis	4	8.5	8.30E−03
Asthma	3	6.4	8.50E−03
Epstein-Barr virus infection	5	10.6	1.30E−02
Graft-versus-host disease	3	6.4	1.50E−02
Intestinal immune network for IgA production	3	6.4	2.00E−02
Malaria	3	6.4	2.10E−02
Osteoclast differentiation	4	8.5	2.30E−02
Yersinia infection	4	8.5	2.30E−02
Efferocytosis	4	8.5	3.30E−02
Influenza A	4	8.5	4.10E−02
Antigen processing and presentation	3	6.4	4.80E−02

**Table 4.** Differentially expressed genes for patient who survived 48 months.

Official gene symbol	Gene name	*P*-value	Fold change
*GZMA*	Granzyme A	.000267	1.897464917
*SELL*	Selectin L	.000267	0.3911819
*TBX21*	T-box transcription factor 21	.000267	2.131373742
*CARD11*	Caspase recruitment domain family member 11 (CARD11)	.000407	2.302650591
*BTLA*	B and T lymphocyte associated (BTLA)	.000842	0.364363786
*LTB*	Lymphotoxin beta	.000842	0.413658981
*THY1*	Thy1 cell surface antigen	.000842	2.564982432
*ATG16L1*	Autophagy related 16 like 1 (ATG16L1)	.001165	1.838070707
*CD38*	CD38 molecule (CD38)	.001165	0.498236368
*IL1B*	Interleukin-1 beta	.001165	4.285055966
*IL4R*	Interleukin 4 receptor	.001165	0.693539465
*CD40*	CD40 molecule (CD40)	.001586	0.41651187
*CD48*	CD48 molecule (CD48)	.001586	0.655830258
*KLRB1*	Killer cell lectin-like receptor B1	.001586	0.408631603
*KLRC1*	Killer cell lectin-like receptor C1	.001586	0.343029278
*CD24*	CD24 molecule (CD24)	.002105	0.374983495
*IFITM1*	Interferon-induced transmembrane protein 1	.002105	0.45969949
*ISG15*	ISG15 ubiquitin-like modifier	.002105	0.276556442
*MERTK*	MER proto-oncogene, tyrosine kinase	.002105	0.602626164
*CCL5*	C-C motif chemokine ligand 5 (CCL5)	.002765	1.850095054
*CD59*	CD59 molecule (CD59 blood group; CD59)	.002765	0.66355009
*CD8A*	CD8 subunit alpha	.002765	2.140738471
*DUSP4*	Dual specificity phosphatase 4	.002765	3.512449806
*NFATC1*	Nuclear factor of activated T cells 1	.002765	0.646466992
*STAT4*	Signal transducer and activator of transcription 4	.002765	1.440531176
*CD22*	CD22 molecule (CD22)	.003565	0.4380309
*CD27*	CD27 molecule (CD27)	.003565	0.430858649
*MX1*	MX dynamin like GTPase 1	.003565	0.265085126
*RORA*	RAR-related orphan receptor A	.003565	1.875284403
*LILRA4*	Leukocyte immunoglobulin-like receptor A4	.004547	0.448119539
*BST1*	Bone marrow stromal cell antigen 1 (BST1)	.005726	0.737976607
*FCER1A*	Fc epsilon receptor Ia	.005726	3.119554745
*FCER2*	Fc epsilon receptor II	.005726	0.221423869
*GZMH*	Granzyme H	.005726	2.249695833
*HLA-DQA1*	Major histocompatibility complex, class II, DQ alpha 1	.005726	3.943122925
*POU2AF1*	POU class 2 homeobox associating factor 1	.005726	0.383596099

## Assessment, analysis, and discussion

**Table:** 

Completion	Study completed
Investigator’s assessment	Limited sample size

The need for novel therapeutic approaches is particularly urgent in PDAC. While immunotherapy is generally easier to tolerate for most patients than chemotherapy, immunotherapy has not, in aggregate, been effective in PDAC. Oncolytic viruses are an appealing area of investigation in pancreatic cancer because pathogens may improve a “cold” tumor-immune microenvironment.

A significant concern related to endoscopic injection of virus is the potential for toxicity, particularly iatrogenic intra-abdominal spread of tumor or infection. No serious toxicities were directly attributable to T-VEC and no direct evidence for the spread of tumor attributed to endoscopy was observed. It was, however, notable that 2 out of 9 patients developed intra-abdominal infections requiring hospital admission. These infections were attributed by the study team to the intra-tumor injections but not to the virus itself, both managed effectively with IV antibiotics. Our results are consistent with the rate of intra-abdominal complications reported with other EUS-administered viruses.^1–5^

Rates of stable disease are difficult to interpret in phase I trials due to selection bias in enrollment, meaning that patients who are very sick are generally not included in trials. Our results with T-VEC are generally similar to prior trials of oncolytic viral therapy in PDAC which demonstrated stabilization of locally injected lesions.^1–3^ Because of the aggressive and highlight lethal nature of PDAC, a 44% stable disease rate would be sufficient to support further investigation of oncolytic viruses, particularly in combination with other immunotherapies. In cutaneous lesions, when oncolytic virus is administered, it is important to carefully perfuse the whole tumor. This was more challenging to do during endoscopic injection, and newer endoscopy or interventional radiology modalities could be useful to maximize the benefits of oncolytic viruses in PDAC.

Although an inflamed immune microenvironment has been proposed to correlate with better outcomes in PDAC, it remains unknown precisely which interventions are needed to decrease local tumor immunosuppression. Further, it is not clear that T-cell infiltration alone is sufficient for tumor regression. Interestingly, while oncolytic viruses, listeria, and radiation induce T-cell infiltration, this infiltration has not yet been proven to correlate with overall systemic response.^4^ T-cell infiltration is therefore insufficient for benefit, and additional systemic interventions may be needed. Observed local control of injected lesions and high rates of stable disease with oncolytic therapy suggest that benefit may be achievable with combination regimens.

There was one exceptional patient who survived for more than 24 months. Analysis of RNA from blood peripheral mononuclear cells (PBMCs) revealed upregulation of transcripts implicated in autoimmune disease and cytokine signaling, consistent with prior reports that favorable changes in the peripheral blood immune milieu may correlate with favorable outcomes in patients with PDAC enrolled in immunotherapy trials.^5^ This observation lends credence to the hypothesis that, although PDAC is largely refractory to immunotherapy, immune parameters have an impact on the clinical course of the disease.

PDAC is an extremely difficult malignancy to treat. The findings presented here demonstrate that intratumoral injection of tumors in patients with advanced PDAC is generally safe. Further trials combining local oncolytic viral therapies with radiation and systemic immunotherapies should be pursued in future studies given the urgent need for effective therapies.

## Data Availability

The data underlying this article will be shared on reasonable request to the corresponding author.
